# Very high-power short duration 90 W/4 s (vHPSD) vs. vHPSD-combined ablation index–guided 50W ablation (hybrid) approach for pulmonary vein isolation in treating atrial fibrillation: have we found the best radiofrequency recipe?

**DOI:** 10.1007/s10840-024-01880-1

**Published:** 2024-08-02

**Authors:** Shaojie Chen

**Affiliations:** Rhythmology/Electrophysiology, Department of Internal Medicine B (Cardiology, Angiology, Pneumology and Internal Intensive Care Medicine), University Medicine Greifswald, Ferdinand-Sauerbruch-Straße, 17475 Greifswald, Germany

Catheter ablation–based electrical pulmonary vein isolation (PVI) is an effective rhythm control strategy in treating atrial fibrillation (AF) [1, 2]. Point-by-point ablation using radiofrequency (RF) energy guided by 3D mapping system has remained the most common energy source for interventional AF treatment to date despite the emergence of alternative technologies using different energy sources and catheter designs.

Procedural success in PVI is the creation of a contiguous, irreversible, transmural, and durable ablation lesion encircling the pulmonary veins, while sparing collateral tissue damage, e.g., the esophagus, the coronary arteries, and the phrenic nerve.

One-year single‐procedure freedom from tachyarrhythmia recurrence after PVI in paroxysmal AF has been reported to be ~ 60% assessed by continuous monitoring using implantable loop recorder [3]. PV electrical reconnection has been recognized as one important factor responsible for AF recurrence after primary PVI [4].

Aiming at enhanced ablation lesion quality, there has been a large interest in using the high-power short duration (HPSD) ablation in the last years. The definition/concept of HPSD varies arbitrarily. Different from the traditional low power long duration ablation (20–40 W/30 s), HPSD mainly refers to “very high-power short duration 90 W/4 s (vHPSD)” or “ablation index–guided HPSD (50 W)” ablation strategies, both represent highly efficient ablation approach [5, 6] and studies on searching the best RF ablation “recipe” for PVI are under ongoing research.

In this issue of Journal of Interventional Cardiac Electrophysiology, Kariki et al. reported on different vHPSD ablation strategies for PVI in paroxysmal AF [7]. This was a prospective, single-center study randomly comparing vHPSD ablation only (vHPSD group) vs. combined vHPSD ablation posteriorly and ablation index (AI)–guided HPSD (50 W) ablation anteriorly (hybrid group) strategies for PVI. The endpoints included procedural characteristics and one year arrhythmia recurrence.

The researchers mainly found that the hybrid approach led to a higher rate of first-pass PVI but similar procedural duration compared to PVI using vHPSD only. Both hybrid approach and vHPSD only approach exhibit high efficacy in terms of freedom from clinical recurrence, and no major complications occurred during the study period. This study supports that the hybrid approach appears to be an optimal ablation strategy to achieve efficient PVI; both hybrid approach and vHPSD only approach seems to be a safe and effective ablation strategy.

The main workflow of the study included: conscious sedation, transseptal puncture, 3D reconstruction of the left atrium using an advanced multipolar mapping catheter (OctaRayTM, Biosense Webster, Inc. CA), and ablation using the QDOT Micro catheter (Biosense Webster, Inc., CA). The temperature controlled QDOT Micro catheter enables ablation in two modes: QMODE + (90 W/4 s) or QMODE (AI-guided 50 W). Other important settings were also provided, e.g., the “target” contact force was 10–25 g, and the inter-lesion distance was 5–6 mm.

Interestingly, compared with the hybrid group the rate of first-pass PVI was significantly lower in the vHPSD group (51% vs. 73%) although vHPSD showed a substantially shorter RF time (424.8 ± 222.4 s vs. 864.5 ± 179.8 s). In patients without first-pass isolation, gaps were more likely to be found in the region of (right) carina in both groups.

First-pass PVI is generally considered a favorable procedural outcome, representing accurate, effective, and contiguous point-by-point lesion set applied in the initial RF encirclement. Moreover, first-pass PVI gives positive impact on procedural efficiency, with less time spent in searching gaps and decreased risk of over-ablation related complications. A low first-pass isolation rate indicates suboptimal initial ablation lesion quality.

Lesion formation and lesion geometry in relation to applied ablation power, duration, contact force, and other parameters remains not fully assessed. A recent preclinical study by Nakagawa et al. demonstrated that tissue temperatures and lesion size (depth, diameter, and volume) were the lowest/smallest for RFs at 90 W/4 s, followed by 50 W/10 s, and the greatest for 30 W/30 s, increasing contact force can significantly increase lesion depth in all three ablation modalities [8]. Moreover, in another recent paper by Pérez et al. found that in low energy setting (360 J) vHPSD (90 W) and HPSD (50 W) produced similar size lesions compared with 30 W/12 s [9].

The “best” RF ablation setting under different conditions remains to be investigated. Nonetheless, the low first-pass PVI in the study from Kariki et al. may be attributed to insufficient contact force, catheter instability, and/or shallow ablation lesion, particularly at the “thick” and “slippery” carina area. Given a fixed power setting, adequate catheter-tissue contact force and catheter stability for a sufficient ablation time are essential for effective lesion formation.

However, both sufficient contact force and catheter stability are sometimes not easy to obtain simultaneously, especially when ablating the “carina” or “ridge” areas. The vHPSD approach, on one hand, using 4-s application time may reduce the chance of catheter instability; on the other hand, instability of the catheter, when it happens, is likely to have a greater impact on lesion formation during such ultra-short application time.

Carefully shortening the inter-lesion distance (≤ 4 mm), using supporting pool, e.g., steerable sheath, and adjustment of respiratory movement may help further improve the lesion quality and increase the rate of first-pass isolation or even PVI durability (Fig. 1).

**Fig. 1:**
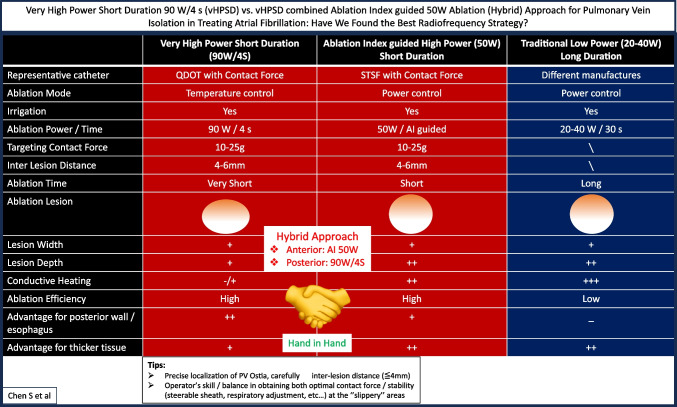
Very high-power short duration 90 W/4 s (vHPSD) vs. vHPSD-combined ablation index–guided 50W ablation (hybrid) approach for pulmonary vein isolation in treating atrial fibrillation: have we found the best radiofrequency strategy?

With respect to the procedural safety, the study by Kariki et al. reported no major complications from both ablation strategies during the study period except one puncture site bleeding in the hybrid group. The safety profile of the vHPSD ablation has been consistently reported by recent studies where low rate of silent cerebral lesion, very low risk of esophageal lesion, phrenic nerve injury, or PV stenosis were found [10].

To conclude, this cohort study by Kariki et al. demonstrated the high procedural efficiency, safety, and similar clinical success rate of both hybrid and vHPSD ablation strategies for PVI in treating paroxysmal AF. Of note, the hybrid approach led to a higher rate of first-pass PVI.

We seem to have found an optimal answer, but we are still on our way to find the best one.

## Data Availability

There are not data available in this article.

## References

[1] Chen S, Pürerfellner H, Ouyang F, Kiuchi MG, Meyer C, Martinek M, Futyma P, Zhu L, Schratter A, Wang J, Acou WJ, Ling Z, Yin Y, Liu S, Sommer P, Schmidt B, Chun JKR. Catheter ablation vs antiarrhythmic drugs as ‘first-line’ initial therapy for atrial fibrillation: a pooled analysis of randomized data. Europace. 2021;23(12):1950–60. 10.1093/europace/euab185. 34405878 10.1093/europace/euab185

[2] Chen S, Pürerfellner H, Meyer C, Acou WJ, Schratter A, Ling Z, Liu S, Yin Y, Martinek M, Kiuchi MG, Schmidt B, Chun KRJ. Rhythm control for patients with atrial fibrillation complicated with heart failure in the contemporary era of catheter ablation: a stratified pooled analysis of randomized data. Eur Heart J. 2020;41(30):2863–73. 10.1093/eurheartj/ehz443. 31298266 10.1093/eurheartj/ehz443

[3] Andrade JG, Champagne J, Dubuc M, Deyell MW, Verma A, Macle L, Leong-Sit P, Novak P, Badra-Verdu M, Sapp J, Mangat I, Khoo C, Steinberg C, Bennett MT, Tang ASL, CIRCA-DOSE Study Investigators. Khairy P Cryoballoon or radiofrequency ablation for atrial fibrillation assessed by continuous monitoring: a randomized clinical trial. Circulation. 2019;140(22):1779–88. 10.1161/CIRCULATIONAHA.119.042622. 31630538 10.1161/CIRCULATIONAHA.119.042622

[4] Chen S, Schmidt B, Bordignon S, Perrotta L, Bologna F, Chun KRJ. Impact of cryoballoon freeze duration on long-term durability of pulmonary vein isolation: ICE re-map study. JACC Clin Electrophysiol. 2019;5(5):551–9. 10.1016/j.jacep.2019.03.012. 31122376 10.1016/j.jacep.2019.03.012

[5] O’Neill L, El Haddad M, Berte B, Kobza R, Hilfiker G, Scherr D, Manninger M, Wijnmaalen AP, Trines SA, Wielandts JY, Gillis K, Lycke M, De Becker B, Tavernier R, Waroux Le Polain De, JB, Knecht S, Duytschaever M. Very high-power ablation for contiguous pulmonary vein isolation: results from the randomized POWER PLUS trial. JACC Clin Electrophysiol. 2023;9(4):511–22. 10.1016/j.jacep.2022.10.039. 36752467 10.1016/j.jacep.2022.10.039

[6] Chen S, Schmidt B, Bordignon S, Urbanek L, Tohoku S, Bologna F, Angelkov L, Garvanski I, Tsianakas N, Konstantinou A, Trolese L, Weise F, Perrotta L, Chun KRJ. Ablation index-guided 50 W ablation for pulmonary vein isolation in patients with atrial fibrillation: procedural data, lesion analysis, and initial results from the FAFA AI High Power Study. J Cardiovasc Electrophysiol. 2019;30(12):2724–31. 10.1111/jce.14219. 31588620 10.1111/jce.14219

[7] Kariki O, Mililis P, Saplaouras A, Efremidis T, Tsetika EG, Martinos A, Girginoudi E, Dragasis S, Letsas KP, Efremidis M. Comparison of very-high power short duration radiofrequency ablation strategies for pulmonary vein isolation in paroxysmal atrial fibrillation. J Interv Card Electrophysiol. 2024 10.1007/s10840-024-01856-1 10.1007/s10840-024-01856-1 38954236

[8] Nakagawa H, Ikeda A, Sharma T, Govari A, Ashton J, Maffre J, Lifshitz A, Fuimaono K, Yokoyama K, Wittkampf FHM, Jackman WM. Comparison of in vivo tissue temperature profile and lesion geometry for radiofrequency ablation with high power-short duration and moderate power-moderate duration effects of thermal latency and contact force on lesion formation. Circ Arrhythm Electrophysiol. 2021;14(7):e009899. 10.1161/CIRCEP.121.009899. 34138641 10.1161/CIRCEP.121.009899

[9] Pérez JJ, D’Angelo R, González-Suárez A, Nakagawa H, Berjano E, d’Avila A. Low-energy (360 J) radiofrequency catheter ablation using moderate power - short duration: proof of concept based on in silico modeling. J Interv Card Electrophysiol. 2023;66(5):1085–93. 10.1007/s10840-022-01292-z. 35796934 10.1007/s10840-022-01292-z

[10] Kottmaier M, Förschner L, Harfoush N, Bourier F, Mayr S, Reents T, Klupp E, Zimmer C, Hadamitzki M, Hendrick E, Krafft H, Lengauer S, Maurer S, Telishevska M, Popa M, Lennerz C, Hessling G, Deisenhofer I. Temperature-controlled high-power short-duration ablation with 90 W for 4 s: outcome, safety, biophysical characteristics and cranial MRI findings in patients undergoing pulmonary vein isolation. J Interv Card Electrophysiol. 2022;65(2):491–7. 10.1007/s10840-022-01146-8. 35748975 10.1007/s10840-022-01146-8

